# PReferentially Expressed Antigen in MElanoma (PRAME): preliminary communication on a translational tool able to early detect Oral Malignant Melanoma (OMM)

**DOI:** 10.7150/jca.82389

**Published:** 2023-02-27

**Authors:** Eliano Cascardi, Gerardo Cazzato, Giuseppe Ingravallo, Miriam Dellino, Carmelo Lupo, Nadia Casatta, Andrea Ballini, Andrea Pacifici, Gaetano Marenzi, Gilberto Sammartino, Eugenio Maiorano, Marco Tatullo

**Affiliations:** 1 Department of Medical Sciences, University of Turin, 10124 Turin, Italy.; 2 Pathology Unit, FPO-IRCCS Candiolo Cancer Institute, 10123 Candiolo, Italy.; 3 Section of Molecular Pathology Department of Precision and Regenerative Medicine and Ionian Area (DiMePRe-J), University of Bari “Aldo Moro”, Bari, 70124, Italy.; 4 Department of Biomedical Sciences and Human Oncology, University of Bari, 70121 Bari, Italy.; 5 Innovation Department, Diapath S.p.A., Via Savoldini n.71, 24057 Martinengo, Italy.; 6 Department of Precision Medicine, University of Campania “Luigi Vanvitelli”, 80138 Naples, Italy.; 7 Department of Oral and Maxillo-Facial Sciences, Sapienza University of Rome, 00195 Rome, Italy.; 8 Department of Neurosciences, Reproductive and Odontostomatological Sciences, University “Federico II” of Naples, via S. Pansini 5, 80131 Naples, Italy.; 9 Department of Translational Biomedicine and Neuroscience (DiBraiN), University of Bari ALDO MORO, 70124 Bari, Italy.; 10 Honorary Senior Clinical Lecturer— University of Dundee, Dundee, Scotland DD1 4HR, UK.; 11 Founder Member of MIRROR—Medical Institute for Regeneration and Repairing and Organ Replacement, Interdepartmental Center, University of Bari ALDO MORO, 70124 Bari, Italy.

**Keywords:** Oral Malignant Melanoma, Early diagnosis, Differential Diagnosis.

## Abstract

Oral malignant melanoma (OMM) has a prevalence less than 1% of all melanomas and it commonly develops on the oral mucosa following a slow and unspecific transformation of unstable melanocytic lesions, often resulting in a diagnostic delay. The marker PReferentially Expressed Antigen in MElanoma (PRAME) seems to be a valid tool to investigate the biological and histological nature of cutaneous melanocytic lesions, but to date its use to characterize pigmented lesions in the oral cavity is largely unexplored. The aim of this study was to create preliminary knowledge on the PRAME expression in OMM, and to compare its expression respect to other dysplastic pigmented lesions of the oral cavity. Interestingly, PRAME has been demonstrated to be reliable in the clinical conditions investigated in our pilot study; in fact, it has clearly differentiated the cases of Melanoma, which showed diffuse and intense positivity (score 6+/7+) to PRAME, from the other melanocytic nevi, which resulted to be mainly negative to PRAME. This means a better differential diagnosis, a reliable early diagnosis and a proper clinical/surgical management of the oncological lesions. In conclusion, PRAME can be a valid qualitative marker for differential diagnosis, not only in cutaneous melanomas, but also in malignant melanoma of the entire head and neck area.

## Introduction

Oral Malignant Melanoma (OMM) is a rare variant of mucosal melanoma 1. OMM epidemiology has recently showed an increased incidence within the Japan population; a predilection for the male sex seems to be generally reported, with a male/female ratio of 2:1 2-4. According to the World Health Organization (WHO) Skin Classification of 2018, OMM is classified among the mucosal melanomas 5, a clinical form that involve about the 40% of the melanomas of the head/neck region 3, 6. Epidemiological studies have highlighted that OMM tends to develop in young subjects 7 and it has a significant preference for the hard palate and gingiva 8.

Although progresses have been made in understanding the etiology of mucosal melanoma, the pathogenesis is not yet fully understood; on the other hand, it is clear that the molecular signature of OMM is different from melanomas originating from surfaces not exposed to ultraviolet (UV) 9. Interestingly, recent studies have highlighted that OMM shows several alterations in the gene expression of CDK4 and Cyclin D1 (CCND1), compared to cutaneous melanoma (CM) which has more mutations in the BRAF and/or NRAS genes 8.

In recent years, a growing interest has involved the marker called PReferentially expressed Antigen in MElanoma (PRAME): it is a member of the cancer testis antigen (CTA) family, which has been studied in the skin melanocytic lesions 10, 11.

Despite its strategic role in several pathogenesis, and although it has been studied in the onset of different neoplasms, there is scarce knowledge about the use of PRAME in the differential diagnosis between OMM and other oral lesions, including the mucosal nevi of the oral cavity.

In this paper, we highlight the most impacting preliminary results of a pilot study carried out on 9 cases of OMM, analyzed with immunostaining for PRAME, and compared with a control group (9 control patients) with benignant nevi developed on the oral mucosa. The main and most important outcome of our comparative study is the preliminary validation of PRAME in the differential diagnosis of similar lesions detected on the intraoral mucosa.

Finally, we critically discuss our results, highlighting the potential clinical perspectives deriving from the early detection of PRAME in OMM, also comparing this approach to the current state of the art.

## Materials and Methods

The cases reported in this pivotal study have been obtained from the archives of the Laboratory of Pathological Anatomy (University of Bari "Aldo Moro"). The authors have used the following searching strategy: the patients were preliminarily clustered by searching the keywords "Melanoma" OR "Malignant Melanoma" AND "Oral". Then, the searching was further narrowed to find “second level” keywords, namely “Nevi” OR “Mucosal Nevi” AND “Oral”. Inclusion criteria were the followings: i. the primary onset of melanoma on the oral mucosa; ii. the absence of other neoplasms in the last 2 years; iii. the absence of a clinical history reporting cutaneous melanoma. Nine patients with the diagnosis of OMM and nine cases reporting intraoral nevi were selected, between the years 2005 and 2019. The clinical and histologic features were investigated, and critically compared, by the same two resident oral pathologists (E.M. and E.C.), and one dermatopathologist (G.C.). All the samples selected and included in this investigation were stored in the same labs and analyzed (5-micron thick sections) with the immunostaining targeted against the recombinant Anti-PRAME antibody [EPR20330] (ab219650). We also decided to compare the PRAME expression in the selected samples with the clinical behavior (benign or malignant lesions) of the patients: more in detail, we investigate the overall percentage of PRAME-positive cells compared to the overall intensity of the immunostaining, by using a cumulative scale considering the amount of tumor cells (0, 1 +, 2 +, 3 +, 4 +) and the related PRAME expression intensity in such tumor cells (0, 1 +, 2 +, 3 +). Sebaceous glands were used as a positive control. Finally, all the reported cases have been further assessed by two independent pathologists, not involved in this study.

## Results

We recruited nine patients diagnosed for OMM (five males and four females); these patients were 65 to 87 years old, with similar baseline clinical conditions.

The OMM onset was diagnosed on hard palate (8 cases) and on gingiva (1 case). All the reported nine cases showed invasive melanomas, three of which were subjected to clinical relapses as melanoma *in situ*, just 1 year following treatment.

Nine (9) patients affected by oral mucosal nevi (MN), 4 males and 5 females, were from 21 to 48 years old. The oral nevi onset was tissue-specific, as follows: on the gingiva (4 cases), on mucous membrane of the cheek (2 cases), on labial mucosa (1), alveolar ridge (1) and vermilion (1). All the reported lesions were safely removed without any relapse after 5 years.

Clinical and pathological features of these patients were summarized in **Tables 1** and **2**.

To better correlate the PRAME expression with its nature (benign, uncertain potential for malignancy or malignant), we categorized PRAME tumor cells' percentage positivity and intensity of immunostaining in a cumulative score, obtained by adding the quartile of positive tumor cells (0, 1+, 2+, 3+, 4+) to the PRAME expression intensity in tumor cells (0, 1+, 2+, 3+). More specifically, we used the following scores for the percentage positivity of tumor cells: 0% (score 0), 1% to 25% (score 1+), 26% to 50% (score 2+), 51% to 75% (score 3+), and 76% to 100% (score 4+). Furthermore, we used a score for intensity by measuring the nuclear immunostaining for PRAME as weak, moderate, or strong (1+, 2+, or 3+, respectively). Sebaceous glands were used as an internal control to confirm the functioning of the PRAME antibody stain. These characteristics of immunoexpression were estimated by both pathologists during the review of the cases.

**Figure 1:**
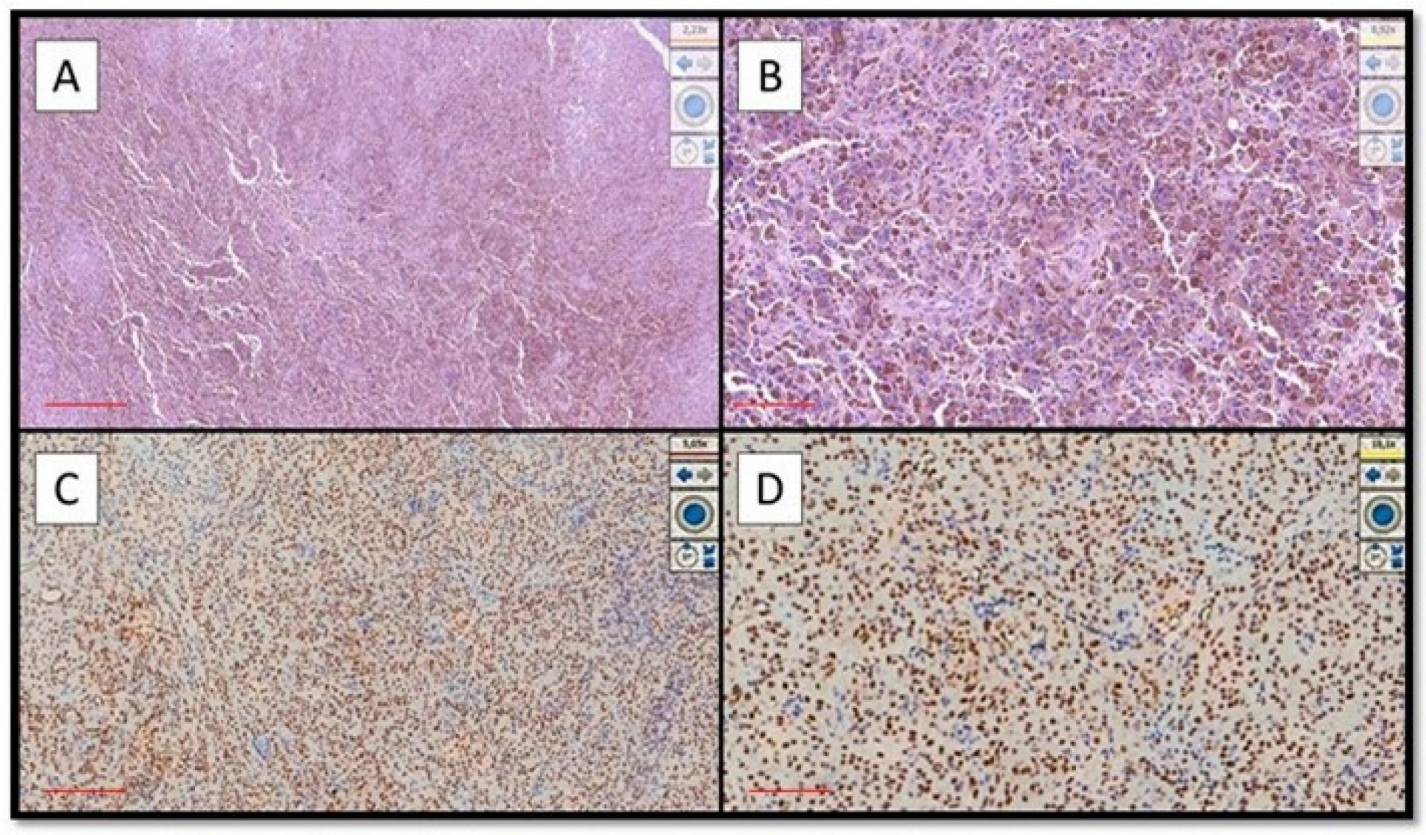
Examples of OMM that were strongly immunopositive for PRAME. **(A)** Histological features of hyperpigmented OMM of the hard palate in a patient with local advanced disease (Hematoxylin-Eosin, Original Magnification 4x, scale bar: 500 µm). **(B)** Another case of pigmented OMM of the gingiva: note the sheets of melanocytic neoplastic cells (Hematoxylin-Eosin, Original Magnification 10x, scale bar: 250 µm). **(C)** Photomicrograph corresponding to the case of (A) showing intense and widespread immunoexpression for PRAME (Immunostaining for PRAME, Original Magnification 4x, scale bar: 500 µm). **(D)** Immunohistochemical preparation of case (B) which also shows strong and widespread positivity of immunostaining for PRAME (Preparation for immunostaining for PRAME, Original Magnification 20x, scale bar: 100 µm).

Eight out of nine cases of OMM were diffusely and intensely positive for PRAME with a total score of 6 + / 7 +; only 1 case was positive for PRAME with a total score of 4+/7+ (Figure 1A-D).

**Figure 2:**
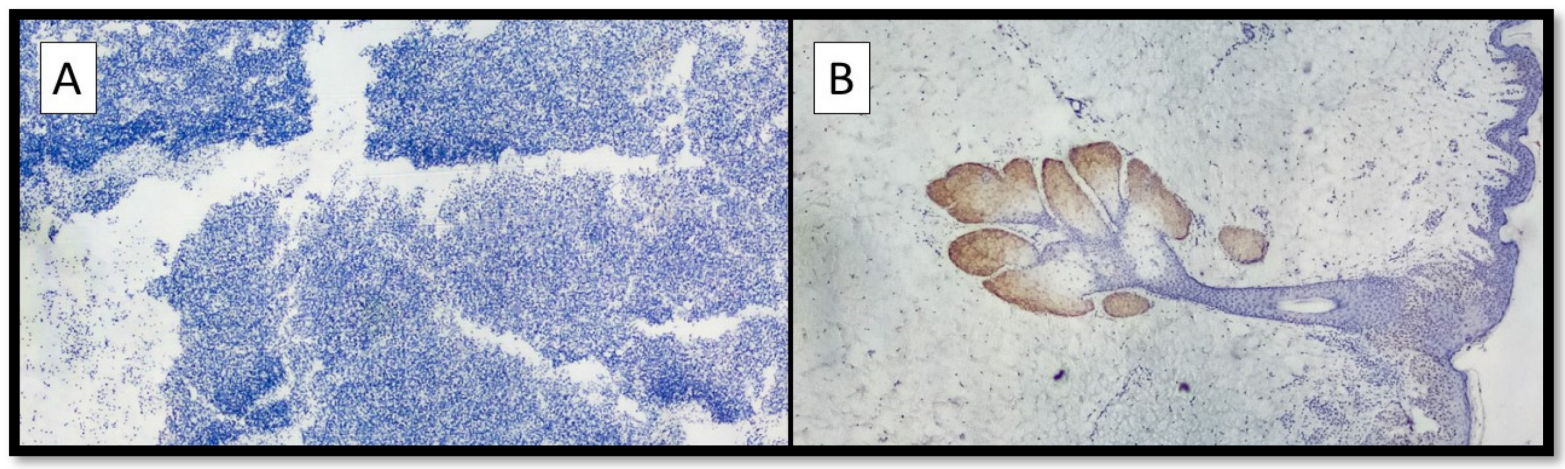
** (A)** Example of benign nevus on oral mucosa: note the almost total negativity for PRAME immunostaining (Immunostaining for PRAME, Original Magnification 10x). **(B)** Representative picture of positive control of PRAME in a sebaceous gland (Immunostaining for PRAME, Original Magnification 10x).

On the other hand, eight out of nine cases of NM were mainly negative for PRAME (Figure 2A) with positive internal control (Figure 2B); more specifically, the lesions showed only focal zones positive PRAME but with a low immunoscore, not exceeding 3 (low expression zone). Only one case was PRAME+ with an immunoscore of 4+/7+ (Figure 1A-D).

## Discussion

OMM represents a very complex and poorly understood variant of mucosal melanoma. Its diagnosis and correct recognition are of extreme importance in order to plan the correct therapeutic-assistance approach for affected patients 4. This lesion can arise in any site of the oral cavity but preferentially it is found on the hard palate and on the gum. Furthermore, OMM can be a consequence of oral melanosis, which is suspected to be the preliminary stage before that the neoplasm invades the underlying tissues (vertical growth phase).

Due to its rarity (0.2% to 0.8% of all melanomas) 12, there are few randomized clinical trials describing OMM in the scientific literature, and there are no clear guidelines on its treatment. In this regard, a recent study 12 has shown that only 447 cases of OMM have been properly described in the literature; 121 cases were diagnosed for metastatic lesions after the primary diagnosis. Often, the clinical presentation of this neoplasm is a challenge, as it is asymptomatic in the early stages; it appears as a macular lesion, irregularly pigmented (brown, black, gray, blue), and sometimes ulcerated in the middle area of the lesion. Therefore, it is difficult to clearly and undoubtedly discriminate the OMM from the mucosal melanocytic nevus and the malignant melanoma; with these premises, it is strongly recommended to remove any suspected pigmented lesion in the oral cavity 13. This surgical indication is further required in consideration of the asymptomatic onset of the OMM; in fact, this silent developmental phase may last for a long period. Moreover, these lesions can appear in different forms (e.g.: pigmented or amelanotic form), often mimicking even the similar benign formations, such as the oral nevi, the melanotic macules or the amalgam tattoos 12-14.

The immunohistochemical characterization of OMMs is well described by the scientific literature: it can be focally positive to the pan-cytokeratin markers (AE1AE3 or MNF116), and the positivity is usually dot-like, as a diffuse and homogeneous staining is not evident in these lesions. On the other hand, OMMs can be strongly characterized by the HMB45, Melan-A and S100 protein staining. Generally, both the clinical and histological differential diagnosis are not always easy to do, as specific forms of OMMs can be negative (or mildly positive) to the classic melanocytic markers, such as Melan-A (MART1) and Human Black Melanoma Antigen-45 (HMB-45) 3, 14. Anecdotally, the marker Ki67 can be useful to discriminate the benign forms of melanocytic tumors from the OMMs 3, nevertheless, Ki67 may be found positive in several other different tissue alterations, thus making this marker not strongly pathognomonic.

In this landscape, the scientific community has highlighted the need to find a novel diagnostic approach that may be useful to the clinicians, to the oral pathologists and to the dermatopathologist, especially for those cases where a complex differential diagnosis is required. In the recent years, several studies have found the reliability of the marker called PReferentially expressed Antigen in MElanoma (PRAME), as a strategic advisor about the potential malignancy of a dermatological pigmented lesion. More in details, after the first paper by Lezcano *et al.* 10, which demonstrated the usefulness and reliability of PRAME in a cohort of pigmented melanocytic lesions, several other authors 15-22 have highlighted the potential (and limits, of course) of this immunostaining technique.

Interestingly, to our knowledge, only 1 paper has been published, by Hovander *et al.* 23, reporting the immunostaining outcomes with PRAME in a cohort of 8 primitive oral melanomas; the authors reported a positivity for PRAME both in *in situ* and in invasive melanomas. These preliminary data have been firstly confirmed by our paper: here, in fact, we have reported the same intensity and distribution of PRAME immunostaining applied to the OMMs; similarly, we have also highlighted the negativity to PRAME in all the mucosal nevi involved in our pilot investigation.

In a recent paper, Vos *et al.* 24 described a single case-report reporting a 54 years-old male patient with a diffuse pigmentation of the hard palate, involving also a small part of the soft palatal mucosa; during the differential diagnosis, in addition to the immunoreactions for Melan-A, HMB-45 and p16, the authors used the PRAME immunostaining that was strongly positive: the histological diagnosis was melanoma *in situ*, further demonstrating the usefulness of PRAME in such lesions.

It is worthy of discussion also the immunomodulatory effects of mesenchymal stem cells (MSCs) in oncological and inflammatory lesions 25; in fact, the anti-inflammatory effect of MSCs is well known in the physiology of inflammation, and such cells have been used as an interesting tool able not only to modulate inflammation on site, but also to ensure reparative processes where required in case of cytotoxic effects induced by some chemotherapies. On the other hand, some studies instead investigated the possible negative implications of stem cells with respect to the prognosis of numerous tumors, including melanocytic ones; in fact, these cells, although endowed with high plasticity and immunomodulatory activity, are also very resistant to chemotherapeutic drugs.

In conclusion, our paper can be considered of particular interest not only because it reports the use of PRAME in the differential diagnosis between intraoral nevi and *in situ* OMM, but also because it first suggests the use of PRAME as a reliable marker, and a kind of surgical guide during the surgical resection of the tumoral margins, improving the chance to successfully achieve the tumor eradication 26, 27.

It is also remarkable that the OMMs are not so common lesions, thus easily subject to wrong diagnosis and clinical misinterpretations; on the other side, there is a concrete limit related to the current difficulty to improve the workflow of all the Pathology laboratories 26 with the anti-PRAME antibody. This study can be considered a milestone in a still long investigation on the pros and cons in PRAME use: a more consistent number of clinical studies may improve the robustness of the entire protocol, creating the robust bases to involve PRAME in the daily workflow of both clinical and histological teamwork.

Differential diagnosis among several benign pigmented lesions of the oral cavity and the OMM represent a challenging topic still little investigated, basically because of the relatively scarce incidence of these lesions. Nevertheless, a late diagnosis could lead to a life-threatening condition for the patient; in fact, both these lesions are usually asymptomatic in the early stages, even showing misleading morphological characteristics. To get a final diagnosis, the physician should require accurate clinical evaluations, as well as specific histological examinations based on the use of different markers that may not have a clear and diriment expression. The use of novel and more specific immunohistochemical markers, able to discriminate among benign pigmented lesions and OMM is undoubtedly a strategic support for a reliable differential diagnosis. Our pilot study has retrospectively and critically assessed the use of PRAME as a valid support in this regard. The authors need to underline that to definitely understand pros and cons of PRAME in OMM diagnosis, further studies are desirable. According to the current scientific literature, this is one of the first studies addressing this clinical challenge: the use of PRAME-supported diagnosis can be a breakthrough in this field, ensuring a more accurate and quick diagnosis. Of course, several trials need to be carried out to strengthen this protocol in the daily clinical practice; our scientific contribute is the first milestone in this direction, and we hope it will be the driving force for future investigations.

## Figures and Tables

**Table 1:** Details of the patients with OMM.

Patients	Age at Diagnosis	Gender	Diabetes	Previous Oncological Lesions (< 2yrs before)	Localization	Histological Diagnosis	Follow-up after three years
1	65	M	No	No	PHP	IM	UN
2	87	F	Yes	Yes	AHP	IM	UN
3	71	F	Yes	No	EP	IM	R
4	73	M	Yes	No	PHP	IM	R
5	81	M	Yes	No	PHP	IM	UN
6	69	M	No	No	PHP	IM	UN
7	77	F	Yes	No	PHP	IM	R
8	76	M	No	No	PHP	IM	UN
9	77	F	No	No	PHP	IM	UN

Legend. PHP (Posterior Hard Palate); AHP (Anterior Hard Palate); EP (Entire Palate); IM (Invasive Melanoma); UN (Unremarkable); R (Relapsed)

**Table 2:** Clinical and pathological features of the patients with mucosal nevi.

Patients	Age at Diagnosis	Gender	Diabetes	Previous Oncological Lesions (< 2yrs before)	Localization	Histological Diagnosis	Follow-up after three years
1	46	M	Yes	No	Cheek	MN	UN
2	21	F	No	No	Gingiva	MN	UN
3	35	M	No	No	Gingiva	MN	UN
4	40	F	No	No	Gingiva	MN	UN
5	33	F	No	No	Cheek	MN	UN
6	29	M	No	No	Gingiva	MN	UN
7	47	M	Yes	No	Labial mucosa	MN	UN
8	41	F	No	No	Vermilion	MN	UN
9	38	F	No	Yes	Alveolar ridge	MN	UN

Legend. MN (Mucosal Nevus); UN (Unremarkable)
